# Impact of cellular proteases on the function of antiviral antibodies

**DOI:** 10.1128/jvi.02141-25

**Published:** 2026-02-19

**Authors:** Mateo Krzypow, Olivier Schwartz, Timothée Bruel, Andréa Cottignies-Calamarte

**Affiliations:** 1 Antiviral Activities of Antibodies Group, Virus and Immunity Unit, Département de Virologie, Institut Pasteur, Paris, France; 2 Virus and Immunity Unit, Département de Virologie, Institut Pasteur, Paris, France; 3 Vaccine Research Institute 838129, Créteil, France; Universiteit Gent, Merelbeke, Belgium

**Keywords:** antibody, proteases, immune escape, virus

## Abstract

Viruses evade antibodies through many mechanisms. While mutation remains the predominant pathway, host proteases can be hijacked upon viral infections and decrease antibody-mediated clearance. Proteolysis can degrade or shed viral antigens to create soluble decoys, induce conformational changes that reduce neutralization, and cleave Fc receptors and complement proteins, hindering Fc-effector antibody functions. This review synthesizes evidence on the interplay between viruses and host proteases to evade antiviral activities of antibodies.

## INTRODUC­TION 

### Mechanisms of viral clearance by antibodies

Viruses and their hosts have been co-evolving in a continual arms race for fitness and survival, respectively (1). In humans, the innate and adaptive arms of immunity intimately interact to control infection. Antibodies (Abs), secreted by certain activated B cells, are an essential portion of the adaptive immune response and are a major pillar in the viral clearance of both enveloped viruses as well as some non-enveloped viruses (1–5). Certain antibodies are developed with the ability, through their fragment antigen-binding region, to bind to viral epitopes and, through a variety of methods (e.g., steric obstruction or changing conformation), result in the neutralization of the target antigen (4).

Antibodies are also a bridge between the adaptive and innate immune responses. Through their fragment crystallizable (Fc) region, antibodies bind to either activators of the complement system or Fc Receptors (FcR) on effector cells, inducing the so-called antibody “effector” or “non-neutralizing” functions, such as complement-mediated cytotoxicity, antibody-dependent cellular cytotoxicity (ADCC), or antibody-dependent cellular phagocytosis (6–8). Together, neutralization and effector function induction place antibodies as correlates of protection across many infections (9–11), as well as at the center of vaccine and therapeutic monoclonal antibody design (2–4, 12).

Apart from complement activation, induction of effector functions depends on the formation of an immune synapse between an antibody-coated target and an effector cell. Globally, this immune synapse depends on Ab density on the target membrane, cofactors within the effector cells (adhesion molecules, signaling molecules, or cofactors such as NKG2D on NK cells), and conditioning by the microenvironment (cytokines, pH, etc.). For complement, completion of the cascade and elimination of viruses and/or infected cells depend on the initial hexamerization of the antibody’s Fc on the target surface and the presence and activity of several inhibitory factors existing within the cascade (7, 11).

Antibody antiviral activities critically depend on site-specific Ab-epitope interaction (13). Thus, reduced recognition/binding of Abs to viral antigens constitutes the major factor in antibody escape. In the context of selective pressure, viruses can evolve to avoid antibody binding through mutations in their genomes, thus creating conformational and/or affinity changes in the Ab-binding site, best exemplified by HIV (14) and, more recently, by SARS-CoV-2 (15, 16). Many other mechanisms exploited by viruses may also lead to a decrease in antibody recognition. Shielding of viral epitopes with glycan sugars lowers antibody binding and dampens their neutralization ability (17). Viruses may also limit or localize the expression of antibody-targeted proteins to reduce epitope availability and thus antibody binding (18). This can be achieved in various ways, including lower expression of viral proteins (e.g., reduced incorporation of envelope protein [Env] within HIV-1 particles [18]), the display of viral proteins in a conformation that reduces antibody recognition (e.g., the closed conformation of HIV-1 Env [19, 20]), or through hydrolytic cleavage by proteases (21–23). While both viral and host proteases are plausible actors in this mechanism, robust data for viral proteases in antibody escape are limited, and evidence for host protease-mediated antibody escape dominates the literature surrounding this topic.

### Proteases in viral immune evasion

Proteases are a broad group of hydrolytic enzymes that irreversibly cleave peptide bonds of proteins (24). Proteases are further subdivided, based on the type of nucleophilic group and/or amino acid involved in the peptide bond cleavage, into six respective classes: serine, cysteine, aspartic, threonine, metalloproteases, and glutamic proteases (25). All but glutamic proteases are found in mammals to date. The nucleophilic group in aspartic and metalloproteases is an externally activated water molecule that attacks the peptide bond (25). The remaining protease classes—cysteine, serine, and threonine—use the respective internal amino acid as a nucleophilic group (25). Since their activity is irreversible, proteases are tightly regulated at the transcriptional level, as well as through the production of zymogen forms and intracellular and/or anatomic compartmentalization (25, 26). Proteases can function as freely secreted enzymes (e.g., secreted digestive enzymes [27]), cell surface-associated enzymes (e.g., transmembrane serine protease 2, TMPRSS2, which is highly expressed in respiratory and gastrointestinal epithelia [28]), or as intracellularly acting enzymes. Intracellular proteases can be further compartmentalized, but not limited, between either free cytosol proteases (e.g., proteasome) or constrained within different organelles of the cells (e.g., furin within the *trans*-Golgi network [29] or cathepsins in endo/lysosomal compartments [30]). Given their involvement in a plethora of human processes such as hemostasis, digestion, mucosal fluids, protein synthesis, cell death, and antigen presentation, proteases can be found throughout the human body (25, 26, 31, 32). Protease content varies with the mucosa type, such as the respiratory tract being particularly rich in Type I transmembrane serine proteases (including TMPRSS2 or human airway trypsin) (32). Notably, viruses can hijack host proteases for their own purposes. For example, coronaviruses frequently employ proteases as a means for cellular entry (33, 34), although the exact reason for this remains unclear. Extensive literature expresses that not only viral proteases (35) but also hijacked host cellular proteases are major contributors to the viral replication cycles, from viral particle formation to envelope/virion maturation and fusion(36–38).

The capacity of viral and hijacked host proteases to impede immune responses is also essential for viral dissemination. Viral proteases have been extensively reported to lower interferon response, such as flavivirus NS3 degrading STING (39) or SARS-CoV-2 NSP5 directly degrading RIG-I (40), among others (35, 39). There are also many examples of host proteases being hijacked by viruses to escape immune response. For instance, HIV-1 Vif and Vpu tag the restriction factors APOBEC and BST2, respectively, for ubiquitin-proteasome-targeted degradation (41). The degradation of factors of the adaptive immune response also exists, with its most striking demonstration being HIV-1 Nef degrading HLA-I by targeting it to the lysosome, preventing the presentation of HIV peptides to cytotoxic CD8 T cells (42). However, the study of how viruses evade adaptive immunity beyond mutations is still emerging. Here, we provide a comprehensive overview of the underappreciated strategies through which viruses co-opt host proteases to subvert humoral immunity.

## HOST PROTEASES DECREASE ANTIBODY NEUTRALIZATION

### Shedding

Cellular proteases can cleave viral antigens from cellular membranes where they are expressed, thereby generating soluble decoys, a process known as “shedding,” which can decrease Ab neutralization. For Ebola virus (EBOV), ADAM17/TACE (tumor necrosis ⍺-converting enzyme, a membranous protease member of the ADAM zinc-dependent metalloproteases family) cleaves the EBOV glycoprotein (GP) ectodomains, releasing an abundant amount of soluble GP subunits as decoys for anti-GP neutralizing Abs (NAbs) to bind to, reducing their neutralization potency (21). In patients infected with Lassa virus, soluble shed GP1 proteins have been found in serum (23) due to glycoprotein complex (GPC) cleavage by cellular subtilase SK-1/S1P, a subtilisin-related serine endoprotease located in the endoplasmic reticulum (43, 44). GPC is cleaved into GP1 and GP2, and a portion of the GP1 is transported to the cell surface, where it is shed (23). Vaccination studies in mice using native/mature GPC and soluble GP1 (cleaved) showed that soluble GP1 induces a non-neutralizing humoral response, which is incapable of targeting the native/mature GPC (45). Authors conclude that cleavage of GPC into GP1 and GP2 and subsequent shedding of GP1 could decoy Ab response (45). The envelope protein of HIV-1 is also subjected to shedding by furin-mediated cleavage of gp160 into gp41 and gp120, which is subsequently secreted (46–48). The secreted gp120 has been found in culture supernatant to act as a moderate decoy for NAbs (49, 50). Soluble Env may also influence Fc-effector functions and possibly promote CD4 T cell depletion through binding to and subsequently inducing ADCC of bystander non-infected CD4 T cells (22). SARS-CoV-1, an intracellularly budding virus, has been found to have its spike protein cleaved by TMPRSS2 (a type I transmembrane serine protease) and shed into the supernatant, acting as a decoy for NAbs and thus decreasing Ab-dependent neutralization (51). It is important to note that most literature exploring viral shedding and antibody functions refers to enveloped viruses, which bud either extracellularly or intracellularly. Whether similar mechanisms of soluble antibody decoy exist in non-enveloped viruses warrants further investigation.

### Conformational changes

Proteases can also influence the conformation of viral proteins through proteolytic cleavage of specific sites, thus altering epitope exposure and potentially reducing Ab binding. In West Nile virus (WNV), host protease furin cleaves the pre-membrane protein (prM) and allows the envelope (E) proteins to change conformation to generate mature virions. This conformational change was found to decrease the binding and neutralizing capacities of NAbs targeting epitopes that are major contributors to the antibody response to WNV infection or vaccination (52). A very close mechanism has been observed with Dengue virus (DENV), where Furin cleavage of the immature precursor membrane protein (prM) into mature membrane protein (M) is not fully effective and thus creates a mix of prM, partially mature M, and mature M proteins, which are incorporated in DENV virions (53, 54). This heterogeneity of M proteins on the viral envelope mitigates the efficacy of anti-prM NAbs, resulting in an increase in the infection of monocytes by antibody-dependent enhancement (ADE) (53, 54). For influenza, proteolytic cleavage of the single-chain precursors (HA0) into HA1/HA2 by either trypsin-like enzymes or furin at the cell surface forms disulfide bridges between HA1 and HA2 to form the activated hemagglutinin (HA) protein. Unlike HA0 (whose low-pH structural changes are reversible), cleaved HA proceeds to irreversible refolding, altering the window during which some neutralizing epitopes are exposed (55). This lowers specific antibody binding and thus the potency of NAbs (56, 57).

Together those results present the cellular protease-mediated escape mechanism of viruses against neutralization, either through decoy generation, decreasing epitope exposure, or through increasing pre-existing ADE.

## HOST PROTEASES REDUCE FC-EFFECTOR FUNCTIONS

### Shedding and lower Ab binding as a consequence of protease activity

Since antibody binding is the initial determinant for effector function efficacy against viruses or infected cells, any of the previously mentioned mechanisms to lower Ab binding may also lead to a decrease in the effectiveness of effector functions. Shed gp120 envelope protein from HIV-1 is reported to prime programmed cell death and suppress T cell response in a CD4-dependent manner (58). Furthermore, another study reports that ADAM17-shed GP from EBOV can induce NK cell death in a caspase-dependent fashion, further reducing ADCC capability (59). Again, for HIV-1, cleavage of gp160 into gp41 and gp120 not only is responsible for shedding but also favors a “closed” conformation, which blocks Abs from binding to critical epitopes for ADCC induction (49, 50, 60).

### Direct targeting of Fcγ receptors

Metzincin metalloproteases, which include ADAM17, are a family of proteases whose activity depends on a conserved methionine in the active site and a zinc ion as a cofactor (61). ADAM17 is known to cleave CD16, the major ADCC-activating Fcγ receptor on NK cells and hence appears as a negative regulator of ADCC by abrogating the Fc-FcR interaction (62). Some studies have shown that viral infections could increase the activity of these regulatory/inhibitory proteases, ultimately protecting infected cells from elimination through ADCC. In chronic hepatitis C infection, the hepatitis C virus (HCV) was found to induce matrix metalloproteinase-9 (MMP9) and ADAM17 mRNA transcription, resulting in an increased ADAM17-dependent cleavage of CD16 on NK cells (63). In HIV-infected individuals, levels of matrix metalloproteinases 1 and 2 (MMP1 and MMP2) transcription were found to be upregulated, decreasing CD16 levels in NK cells, a state which would be reversed upon MMP inhibition (64). This decrease in CD16 most likely translates into lower NK cell responsiveness to Ab-coated cells and a general loss of ADCC function during HIV infection. HIV-1 infection has been reported to promote MMP-mediated shedding of natural killer group 2 member D ligands (NKG2DL) to the bloodstream, causing a decrease in NKG2D expression by NK cells (65). As the NKG2D-NKG2DL axis has been proven to be involved in NK cells ADCC, these mechanisms would participate in HIV escape from ADCC (66). During human herpes virus (HHV) 6A or 6B infection, it has been found that at least two viral proteins (among which is U21) reroute NKG2DLs, such as ULBP3 (an essential activator ligand of NKG2D), to the proteasome (67, 68). This degradation and loss of NKG2DL expression accounts for reduced NK cell-mediated cytotoxicity against infected cells. In HHV-6, Ab responses have only been found to moderately neutralize infection, while cellular responses appear to be more relevant to the control of infection (69). Thus, impairment of the NKG2D-NKG2DL axis may contribute to a lower clearance of HHV-6 infected cells.

### Evasion of complement activation

Viruses can also hijack cellular proteases to disrupt the host complement system. The complement pathway can be activated by Abs through the classical pathway (C1q recognition of Abs) and by the lectin pathway (MBL recognition of pathogen-specific sugars) (70). Astroviruses are non-enveloped viruses that encode a coat protein (CP) that has been found to bind to C1q (an activator of the C1r and C1s proteases that initiate the classical pathway of complement activation) and MBL. The binding of CP to C1q and MBL was found to inhibit complement activation through displacing the C1r/C1s serine protease tetramer from C1q and through blocking residues necessary for the association of MBL to its serine proteases (71), overall allowing infected cells to evade the complement system. While this paper did not directly investigate downregulation of complement activation against Ab-coated infected cells, it did find the overall decrease in Ab-induced complement activation using rat sera and sheep erythrocytes in a CP dose-dependent manner (71). A more common mechanism employed to escape complement is through the cleavage of critical proteins downstream in the complement cascade, which, under normal circumstances, would ultimately lead to the formation of the membrane attack complex (MAC) and thus cause lysis of the cell or viral membrane (70). In flaviviruses, NS1 from WNV, DENV, and yellow fever virus has been found to bind to C4 binding protein (C4BP) to recruit and activate the protease Factor I, a critical negative regulator of the complement system. Activated Factor I cleaves C4b, a pivotal actor in complement activation, thus protecting flaviviruses against complement-mediated neutralization (72, 73). The NS1 protein of WNV was also found to bind to Factor H, a cofactor to Factor I, thereby increasing Factor I’s ability to degrade C3b in solution. NS1, bound to Factor H, was also found to inactivate the C3bBb convertase (a serine protease that aids in C3b deposition on cell surfaces), thus modulating C3b deposition and inhibiting formation of C5-b9 MAC on cell surfaces (74). In Kaposi’s sarcoma-associated herpesvirus (KSHV), infected cells produce soluble and cell-associated KSHV complement control protein (KCP), which recruits Factor I to cleave C3b and C4b, once again lowering complement-mediated neutralization (75) and infected cell clearance (76). Finally, the vaccinia, variola, and monkeypox viruses dampen alternative complement activation by the interaction of viral protein VCP (vaccinia virus complement control protein), SPICE (smallpox inhibitor of complement enzymes), or MOPICE (monkeypox inhibitor of complement enzymes) with Factors I and H to promote C3b and/or C4b degradation (77–79). Given the predominant role of complement in the neutralization of poxviruses (80, 81), these mechanisms appear to be key for dissemination. Even though all reported mechanisms of hindering complement activation have not been strictly shown in the context of antibodies, general inhibition of complement effectiveness would impair antibody-dependent complement pathways.

Collectively, viruses have co-opted different strategies to evade effector functions either by altering ADCC immune synapse quality, NK cell viability, or by displacing initiator proteases and/or recruiting regulators of components of the complement system and thus suppressing it, thereby shielding infected cells from humoral immune attack.

## DISCUSSION

**Fig 1:**
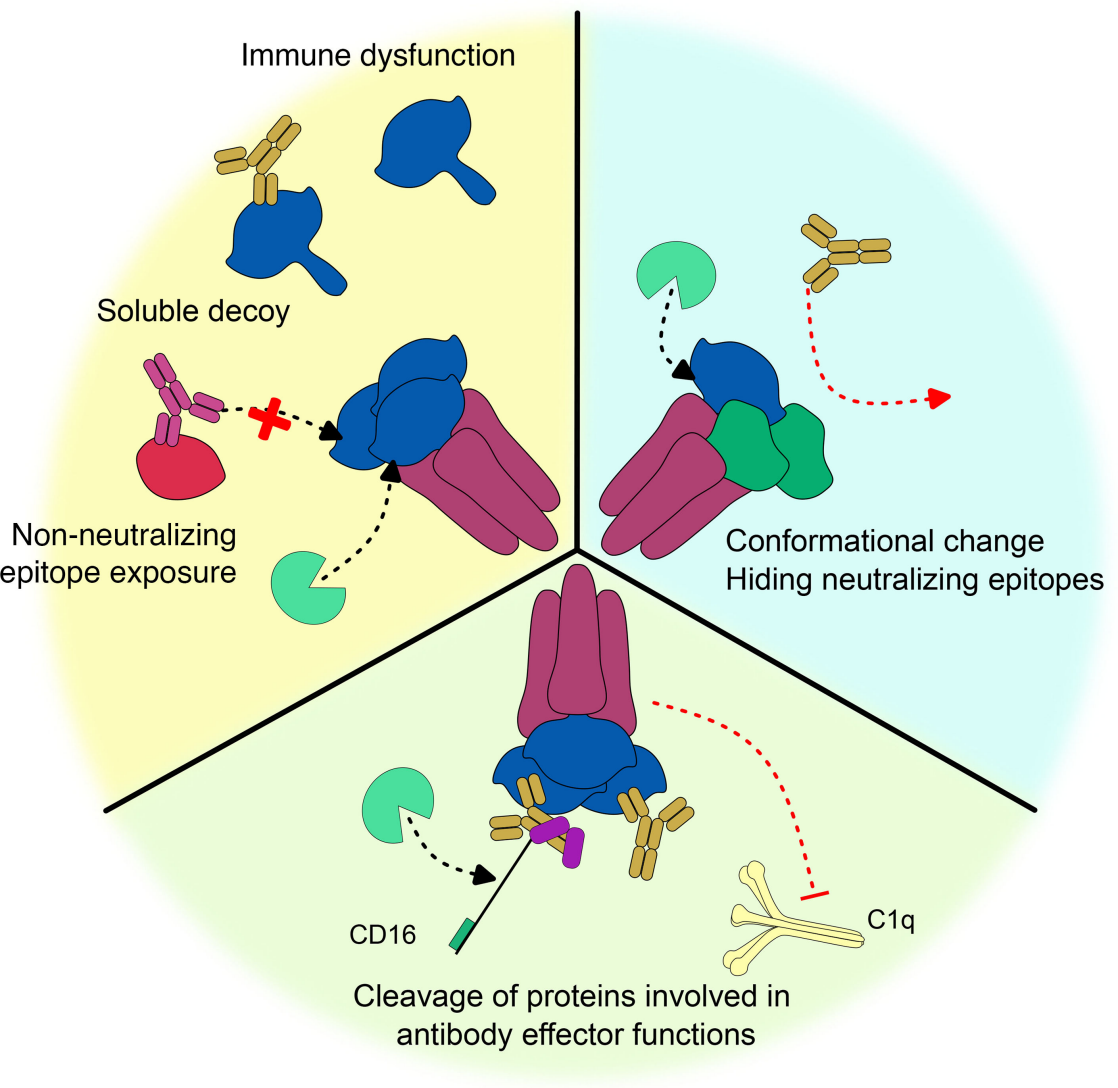
Summary of host protease subversion by viruses to escape the antiviral activities of antibodies. Yellow background shows the role of soluble decoys in luring neutralization, promoting immune dysfunction, or revealing epitopes, which generate non-neutralizing antibodies. Blue background illustrates how protease-induced conformational changes modify/hide neutralizing epitopes. Green background summarizes how viral subversion of host proteases lowers effector functions against viruses or infected cells.

Antibody effectiveness against viruses is not only modulated by viral antigen sequence, glycan shielding, or tight moderation of viral protein expression but also by host protease activity at various stages of the viral cycle and through numerous pathways (Table 1). In this review, we identified three reoccurring host-protease-mediated routes of antibody escape, as represented in Fig. 1, including (i) shedding of viral antigens, which can generate soluble decoys not only reducing available NAbs to bind to infected cells but also inducing off-target ADCC; (ii) induction of conformational changes, which can mask or reduce epitope accessibility and neutralization/antibody binding; and (iii) direct impairment of effector pathways, including FcR cleavage and co-opting complement regulators to limit C3b/C4b deposition.

**TABLE 1:** Mechanisms through which proteases mediate evasion of antibody response

Virus	Host protease	Viral actor, mode of action	Mechanism	Reference(s)
EBOV	ADAM17	GP, cleaved	Shed GP decoys neutralization; induces NK cell apoptosis	(21, 59)
Lassa virus	SK-1/S1P	GPC, cleaved	Shed GPC decoys neutralization; promotes non-neutralizing response	(23, 43, 45)
HIV-1	Furin	gp120, cleaved	Shed gp120 decoys neutralization; immune dysfunction; ADCC of bystander non-infected CD4 T cells; promotes “closed” conformation of Env resistant to ADCC	(22, 46–50)
SARS-CoV-1	TMPRSS2	Spike, cleaved	Shed spike decoy neutralizing Abs	(51)
WNV	Furin	prM, cleaved	E conformational change; lower neutralization	(52)
Dengue virus	Furin	prM, cleaved	Heterogeneous M maturation on viral particles lowers anti-prM NAb efficacy; increased infection via ADE	(53, 54)
Influenza	Trypsin-like or furin	HA0, cleaved	Altered epitope exposure; lower NAb potency	(55–57)
HCV	MMP9 and ADAM17	Unknown, transcriptional	CD16 cleavage on NK cells reduces NK cell ADCC	(63)
HIV-1	MMP1 and MMP2	Unknown, transcriptional	Upregulation of MMP decreases CD16 levels and ADCC; shedding of NKG2DL ligands reduces NKG2D expression by NK cells	(64, 65)
HHV-6A and HHV-6B	Proteasome	U21, docking to proteases	Reroutes NKG2D ligands to the proteasome,reducing NK cell-mediated cytotoxicity	(69)
Astroviruses	Binding to initiator proteases (C1q, MBLs)	Coat protein, docking to proteases	Inhibits C1q and MBL by blocking activatory residues, thereby decreasing complement activation	(71)
WNV, DENV, yellow fever virus	Factors H and I	NS1, docking to proteases	Recruits and activates Factor I to cleave C4b and C3b; protects against complement-mediated neutralization	(72–74)
KSHV	Factor I	KCP, docking to proteases	Recruits Factor I to cleave C3b and C4b; protects against complement-mediated neutralization	(75, 76)
Vaccinia, variola, monkeypox	Factors H and I	VCP, SPICE, MOPICE, docking to proteases	Acts as a cofactor for Factors I and H to cleave C3b and C4b; protects against complement-mediated neutralization	(77–79)

Overall, viral hijacking of host proteases constitutes another yet underappreciated axis of viral escape from antibodies. Considering the magnitude by which cellular proteases are intertwined within innumerable processes within the human body, there could be countless other unelucidated ways by which viruses take advantage of cellular proteases. Furthermore, while the most obvious protease-mediated escape mechanism from effector function is the cleavage of either antiviral Abs or FcR proteins, impairment of any of the proteins involved in effector functions and their respective activation cascades could result in devastating effects on the host’s ability to eliminate infected cells (82). Proteases possess natural inhibitors encoded by the host genome, such as serpins or protease-activated receptors. It remains to be elucidated how virus-induced changes in protease activity are counteracted by these protease inhibitors. Despite the seemingly conceptual straightforwardness, these mechanisms are methodologically difficult to dissect. The effects are pleiotropic, case specific, and intertwined with essential steps of cellular function. Many of these mechanisms may also have a plethora of secondary effects that have not been measured due to the limited scope of *in vitro* studies. Such studies may underrepresent important factors such as tissue-protease gradients, mucus-barrier chemistry, and/or long-range effects that may be prevalent in patients. While *in vivo* studies would broaden the range of measurement, they come with their own many complications and difficulties.

The implications of viruses evolving to hijack host proteases to evade host immune systems necessitate further thorough investigation, as therapeutic counteractions targeting cellular components are difficult to enact due to the potential for major adverse side effects. As previously mentioned, cellular proteases have diverse functions throughout the human body that, if impeded by a therapeutic protease inhibitor, for example, could spell unintended effects and thus necessitate caution. To circumvent the potential adverse effects of current protease inhibitors, several studies have developed nanobodies targeting specific proteases such as furin or trypsin-3 (83, 84). In another study, a non-cleavable peptide conjugated to a nanobody generates a highly potent trypsin inhibitor, which suppresses H7N7 influenza replication *in vitro* (85). However, those approaches do not enable the specific targeting of infected cells. Research on viral and cellular proteases has also raised timely questions, such as to what extent viruses could evolve to use host proteases to disrupt host functions, and to what extent therapeutics could counter such evolutions. For example, many bacteria have been found to express IgG-cleaving proteases, such as *Staphylococcus aureus* coding for glutamyl endopeptidase I (Gluv8), a serine protease (86). Interestingly, herpes simplex virus 1 has been reported to code for a viral FcR, gI, and gE, which have been found to sequestrate IgG from acting on the virus and on infected cells (87). It is still unknown if other viruses could evolve to hijack host proteases to achieve the same function. In fact, it has been observed that matrilysin (MMP-7, naturally expressed in epithelial cells), stromelysin-1 (MMP-3, naturally expressed in a variety of cells, such as fibroblasts, endothelial, and epithelial cells [88]), and other MMPs (-2,-12, and -13) are able to cleave all subclasses of human IgG (89, 90). MMP-2, MMP-3, MMP-7, MMP-12, and MMP-13 have been found to be upregulated in certain tumor cells (89, 90). This could be a mechanism by which tumor cells escape effector functions (89, 90). It raises precedence to further investigate current viral and host protease interactions with regard to the therapeutic potential of antagonizing such interactions. Of note, TMPRSS2 inhibition by camostat failed to treat COVID-19 during clinical trials (91, 92). This indicates that, even if promising, there is much more to learn about the interplay between viruses and host proteases to efficiently target this axis for therapeutic purposes.

## FURTHER DIRECTION AND LIMITATIONS

Not only is there a great diversity in participating viruses but also in the type of proteases engaged. Even though the viral proteins involved in the repurposing of cellular proteases are not identified in every study, it remains a clear escape mechanism for which the identification of viral players would reinforce further pillars of study. Of note, we could not find studies reporting such an effect for antibody-dependent cell phagocytosis or viral phagocytosis, though it remains conceptually possible in a similar manner as we report here (e.g., proteolytic cleavage of CD32 rather than CD16). We could not find any studies reporting the incorporation of cellular proteases into virions, thus exerting the described antibody-evasive function in a virion-associated manner. The cellular proteases involved in Ab escape span multiple classes from furin to ADAMs, MMPs, cathepsins, etc., and it appears that most of the studies have identified the involvement of serine- and metalloproteases. Whether this is a general trait for protease-mediated Ab escape or is virus-dependent remains to be elucidated.

In the cases for viruses that do use cellular proteases to evade effector functions, not much literature explores their effect on antibody-based vaccine efficacy. Similarly, while it is known that some viruses employ cellular protease-mediated immune evasion, possible effects on pathophysiology remain unexplored. The rise of multiomics and spatial biology, as well as the development of class-specific pharmacological protease inhibitors, should allow future studies to identify the proteases involved in any given viral cycle faster and more accurately. There is much to be elucidated in the realm of the extent of the implications of such immune-escape functions of cellular proteases, whether direct or indirect. Many questions and knowledge gaps persist, and further studies are needed to fully elucidate the role of proteases in antibody-mediated viral clearance in both disease progression and therapeutic contexts.
